# Effect of Na, K and Ca Salts on Growth, Physiological Performance, Ion Accumulation and Mineral Nutrition of *Mesembryanthemum crystallinum*

**DOI:** 10.3390/plants13020190

**Published:** 2024-01-10

**Authors:** Astra Jēkabsone, Andis Karlsons, Anita Osvalde, Gederts Ievinsh

**Affiliations:** 1 Department of Plant Physiology, Faculty of Biology, University of Latvia, 1 Jelgavas Str., LV-1004 Rīga, Latvia; astra.jekabsone@lu.lv; 2 Institute of Biology, University of Latvia, 4 Ojāra Vācieša Str., LV-1004 Rīga, Latvia; andis.karlsons@lu.lv (A.K.); anita.osvalde@lu.lv (A.O.)

**Keywords:** chlorophyll, chlorophyll *a* fluorescence, ion accumulation, *Mesembryanthemum crystallinum*, mineral nutrition, obligatory halophytes

## Abstract

*Mesembryanthemum crystallinum* L. is an obligatory halophyte species showing optimum growth at elevated soil salinity levels, but the ionic requirements for growth stimulation are not known. The aim of the present study was to compare the effects of sodium, potassium and calcium in the form of chloride and nitrate salts on the growth, physiological performance, ion accumulation and mineral nutrition of *M. crystallinum* plants in controlled conditions. In a paradoxical way, while sodium and potassium had comparable stimulative effect on plant growth, the effect of calcium was strongly negative even at a relatively low concentration, eventually leading to plant death. Moreover, the effect of Ca nitrate was less negative in comparison to that of Ca chloride, but K in the form of nitrate had some negative effects. There were three components of the stimulation of biomass accumulation by NaCl and KCl salinity in *M. crsytallinum*: the increase in tissue water content, increase in ion accumulation, and growth activation. As optimum growth was in a salinity range from 20 to 100 mM, the increase in the dry biomass of plants at a moderate (200 mM) and high (400 mM) salinity in comparison to control plants was mostly due to ion accumulation. Among physiological indicators, changes in leaf chlorophyll concentration appeared relatively late, but the chlorophyll *a* fluorescence parameter, Performance Index Total, was the most sensitive to the effect of salts. In conclusion, both sodium and potassium in the form of chloride salts are efficient in promoting the optimum growth of *M. crystallinum* plants. However, mechanisms leading to the negative effect of calcium on plants need to be assessed further.

## 1. Introduction

The studies of the physiological and molecular mechanisms of plant salt tolerance has attracted large scientific interest in the recent decades. One of the reasons is related to the desire to reduce plant production losses caused by environmental salinization, and an understanding of salt tolerance could help greatly. In this sense, salt-tolerant wild plant species are interesting models for studies aimed at uncovering plant salt adaptation mechanisms [1,2]. On the other hand, there is interest in studying the properties of salt-tolerant plants in relation to their possible practical uses [3,4,5,6,7].

*Mesembryanthemum crystallinum* L. is an annual succulent species of Aizoaceae, native to the Namibian desert of southern Africa, and has been introduced in coastal areas of another continents [8]. The plant has unique adaptive properties, allowing it to withstand high substrate salinity and episodes of water shortages, including the presence of epidermal bladder cells on shoots that act as water and salt reservoirs as well as an inducible transition from a C_3_- to a Crassulacean acid metabolism (CAM)-type of photosynthetic carbon fixation [9]. The species has emerged as a model plant in studies aimed at understanding the molecular mechanisms of salinity tolerance [10,11,12,13,14] as well as in respect to its use as a halophytic crop species for salinized soils or in cultivation systems using seawater [15,16,17,18,19,20,21,22]. In addition, its heavy metal tolerance and accumulation potential have been studied [23,24,25,26].

*M. crystallinum* has been sometimes classified as a “facultative halophyte” [18,27,28], but it is not reasonably clear what the basis of this designation was. It is possible that the designation comes from earlier studies, in which *M. crystallinum* was labeled an “inducible halophyte”, and it was even stated that “As a facultative halophyte, the ice plant undergoes a set of stress-inducible physiological and biochemical changes that allow it to adjust and maintain cell viability and turgor, conserve water and continue growth during extreme salt stress” [29]. In contrast, *M. crystallinum* plants usually show optimum growth at clearly elevated rootzone salinity (50 to 200 mM NaCl) [8,13,20]. Therefore, it can be categorized as an obligatory halophyte species [20]. 

Insights into the molecular mechanisms of the adaptations of *M. crystallinum* plants have revealed several unique features related to sustained plant growth in high salt environments. At the level of the regulation of the salt adaptation reactions of *M. crystallinum* plants, a positive role of the transcription factor McHB7 has been shown [14]. In addition, the micro-RNA-mediated post-transcriptional regulation of Na^+^ distribution had an important role in its adaptation to salinity, as Na^+^ was used as an osmoticum for cell expansion, but excess Na^+^ was stored in epidermal bladder cells [12]. At the same time, the bladder cells had unique cell-type-specific transcriptomes with important roles in the whole-plant salt adaptation capacity of *M. crystallinum* plants [11]. 

It is argued that the increased growth of obligatory halophytes under salinity results from the ionic effects instead of osmotic effects of salinity [13]. However, particular ionic requirements for the salinity-induced optimum growth of obligatory halophytic plants have not been broadly studied, with only some exceptions [30,31]. Instead, usually, NaCl has been used as a salinity agent. However, given the high chemical similarity between Na^+^ and K^+^, it seems to be logical to propose that both ions can act as growth-stimulating agents for obligatory halophyte species. In general, for relatively salt tolerant plants, the growth responses to Na^+^ and K^+^ salts are similar [32], but in some cases, K^+^ salts seem to be even more toxic at higher concentrations [33]. In a series of experiments, it was shown recently that the anionic component of salts is a significant determinant of salinity tolerance as well as ion accumulation potential for halophytes [32,34,35,36].

Alterations in mineral uptake and the subsequent disruption of mineral balance in plant tissues have been cited among the reasons for the negative effects of salinity in plants [37]. Even relatively salt-tolerant plants show significant changes in the composition of minerals at high salt concentrations [38,39]. It is still not clear whether salinity-related changes in mineral nutrition can lead to direct detrimental effects on plants [37]. Only a limited amount of information is available on what changes in mineral nutrition status occur in obligatory halophytes during salt-induced growth [31,40]. 

The measurement of chlorophyll *a* fluorescence by non-destructive means during plant growth and development can be used for the prediction of the physiological state of plants even before any visible morphological changes have appeared [41,42]. In addition, a detailed analysis of changes in different fluorescence parameters allows one to trace the influence of the suboptimal environmental factor at different structural levels of the photochemistry of photosynthesis [43,44]. The use of high-resolution analyses of the induced fluorescence kinetics has been especially useful in the assessment of salinity responses in halophytes [1].

The aim of the present study was to compare the effects of sodium, potassium and calcium in the form of chloride and nitrate salts on the growth, physiological performance, ion accumulation and mineral nutrition of *M. crystallinum* plants in partially controlled conditions. In particular, it was hypothesized that both sodium and potassium in the form of chloride salts will be sufficient and necessary factors for the optimum growth of *M. crystallinum* plants. 

## 2. Materials and Methods

### 2.1. Plant Material, Establishment and Cultivation Conditions

Seeds of *Mesembryanthemum crystallinum* L. were purchased from Jelitto Perennial Seeds, Schwamstedt, Germany. The seeds were sown in autoclaved commercial garden soil (Biolan, Eura, Finland) mixed with sterile deionized water in closed 1 L tissue culture containers. The containers were kept in a MLR-352H plant growth cabinet (Sanyo Electric, Osaka, Japan) and exposed to photoperiodic light (16/8 h light/darkness, photosynthetically active radiation with a photon flux density of 40 µmol m^−2^ s^−1^) with day/night temperatures of 25/15 °C. When seedlings developed their first two true leaves, they were individually transplanted into 250 mL plastic containers filled with a mixture of garden soil (Biolan, Eura, Finland) and quartz sand (Saulkalne S, Saulkalne, Latvia (4:1, *v*/*v*)). The containers were placed in 48 L plastic boxes closed with lids and placed in the experimental automated greenhouse (HortiMaX, Maasdijk, The Netherlands) for gradual accommodation. Lighting was provided by natural daylight and supplemental artificial lighting from Master SON-TPIA Green Power CG T 400 W (Philips, Amsterdam, The Netherlands) and Powerstar HQI-BT 400 W/D PRO (Osram, Munich, Germany) lamps (16/8 h light/darkness, photon flux density of photosynthetically active radiation of 380 mol m^−2^ s^−1^ at the plant level). Day/night temperatures were 25/15 °C, and the relative humidity was 60–70%. Soil moisture was measured daily using an HH2 meter equipped with a WET-2 sensor (Delta-T Devices, Burwell, UK) and was kept at 50–60% using deionized water. 

### 2.2. Treatments and Physiological Analysis

Salinity treatments were started at the juvenile stage (four true leaf pairs) of the plants. Two separate experiments were performed. In the first experiment, *M. crystallinum* plants were randomly divided into 13 treatments, with five plants per treatment as biological replicates. Plants were gradually treated with a NaCl, KCl, CaCl_2_, NaNO_3_, KNO_3_ or Ca(NO_3_)_2_ solution within two weeks until the amount of the respective anion (Cl^−^ or NO_3_^−^) in substrate reached 100 or 200 mol L^−1^. Control plants were treated with deionized water. In the second experiment, *M. crystallinum* plants were randomly divided into 16 treatments, five plants per treatment as biological replicates. Plants were gradually treated with a NaCl, KCl or CaCl_2_ solution within four weeks until the amount of Cl^−^ in the substrate reached 20, 50, 100, 200 or 400 mol L^−1^. Control plants were treated with deionized water.

In Experiment 1, the leaf chlorophyll concentration and chlorophyll *a* fluorescence analyses were performed one week after the last treatment. In Experiment 2, the leaf chlorophyll concentration and chlorophyll *a* fluorescence analyses were performed three times during plant cultivation: on the day of the last treatment, and one and two weeks after the last treatment. The leaf chlorophyll concentration was measured using a CCM-300 chlorophyll meter (Opti-Sciences, Hudson, NH, USA). The two largest leaves from all plants per treatment were measured. The chlorophyll *a* fluorescence was measured in the largest leaf from all plants per treatment. Leaves were dark-adapted for at least 20 min by designated leaf clips, and induced fast fluorescence was measured using a Handy PEA fluorometer (Hansatech Instruments, King’s Lynn, UK).

A fluorescence data analysis was performed by PEA Plus software (v. 3.11, Hansatech Instruments, King’s Lynn, UK). A number of parameters derived from the fast fluorescence induction curve were used for the analysis [42,45]. F_v_/F_0_, calculated as (F_m_ − F_0_)/F_0_, is considered to reflect instant photochemical activity at the donor side of photosystem II. The Performance Index (representing a multiparametric entity) is used as a relative indication of sample vitality and can have different types of expression. Thus, Performance Index Inst combines three function-related parameters (the trapping of absorbed excitons, electron transport between the photosystems, and the reduction in end-electron acceptors). Performance Index Total includes information on the status of both photosystem II and photosystem I in addition to characterizing the electron flow between the two systems, which is on an absorption basis. 

### 2.3. Termination of the Experiments and Measurements

Both experiments were terminated four weeks after the full treatment. At the time of termination, the development of side shoots with secondary leaves had started, indicating a transition to the adult phase [9]. 

For Experiment 1, sequential leaf pairs were collected and handled separately (Figure S1). For Experiment 2, plant shoots were individually separated in leaves and stems. Roots were carefully washed from any substrate particles. The plant material was weighed before and after drying in an oven at 60 °C until a constant mass was reached. The water content was calculated as g H_2_O per g dry mass. The dry plant material was used for the analysis of soluble ions (Experiment 1) or for the measurement of the total concentration of mineral elements (Experiment 2).

For the analysis of soluble ions, plant material was crushed by hand, and a homogenous tissue sample (0.2 g) was ground using a mortar and pestle to a fine powder. Deionized water (10 mL) was added and stirred with the pestle for 1 min. The homogenate was filtered through a nylon mesh cloth (No. 80) and used for the measurement of electrical conductivity (EC) using LAQUAtwin B-771 conductivity meter, Na^+^ concentration using LAQUAtwin B-722 compact meter, K^+^ concentration using a LAQUAtwin B-731 compact meter, and Ca^2+^ concentration using a LAQUAtwin B-751 compact meter (Horiba, Kyoto, Japan). Six tissue samples were independently measured for each salt/leaf pair combination.

An analysis of the total concentration of Na^+^, Cl^−^ and mineral nutrients in plant leaves was performed on the dried and mineralized tissue samples, as described previously [46]. The levels of Na, K, Ca, Mg, Fe, Cu, Zn and Mn were estimated using an Agilent 4200 microwave plasma atomic emission spectrometer (Agilent Technologies, Santa Clara, CA, USA), and P levels were analyzed by colorimetry with ammonium molybdate in an acid-reduced medium using a Jenway 6300 spectrophotometer (Cole-Palmer Instrument Company, St. Neots, Cambridgeshire, UK), but values of Cl were obtained by AgNO_3_ titration via the distilled water extraction of plant ash. All analyses were performed in triplicate using representative tissue samples from individual biological replicates.

For the analysis of the relative contribution of ion accumulation in salinity-stimulated biomass accumulation, the increase in the content of Na^+^ and Cl^−^ (in NaCl-treated plants) as well as content of K^+^ and Cl^−^ (in KCl-treated plants) was expressed on an individual plant basis. The increase in dry organic biomass was calculated by subtracting the ion accumulation from the total dry mass gain on an individual plant basis. 

### 2.4. Data Analysis

The results were analyzed using KaleidaGraph software (v. 5.0.6, Synergy Software, Reading, PA, USA). The statistical significance of differences was evaluated by one-way ANOVA using the Tukey post hoc test with honestly significant difference (*p* < 0.05). Principal component analysis and heat map generation were performed using ClustVis (http://biit.cs.ut.ee/clustvis/, accessed on 15 December 2023), a freely available web program [47]. Hierarchical clusters were generated using the average linkage method with correlation distance.

## 3. Results

### 3.1. Experiment 1: Comparison of Six Different Salts

**Figure 1:**
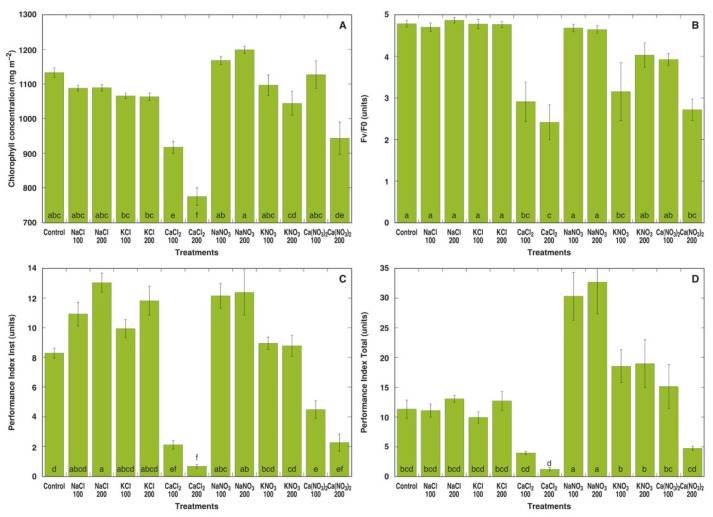
Effect of different salts on leaf chlorophyll concentration (**A**), chlorophyll *a* fluorescence parameters F_v_/F_0_ (**B**), Performance Index Inst (**C**) and Performance Index Total (**D**) of *Mesembryanthemum crystallinum* plants one week after the last treatment. Numbers on x axis indicate respective salt concentration in soil (mM). Data are means from 10 independent measurements for chlorophyll concentration and five independent measurements for chlorophyll *a* fluorescence parameters ± SE. Different letters indicate statistically significant differences according to the Tukey HSD test (*p* < 0.05).

In the first experiment, *M. crystallinum* plants were treated with sodium, potassium and calcium in the form of chloride and nitrate salts at two concentrations each (equalized in respect to the anion concentration in the soil). Already one week after the full treatment with different salts, pronounced morphological differences in *M. crystallinum* plants between various treatments were evident (Figure S2A). Plants treated with NaNO_3_ or KNO_3_ showed the most vigorous growth, while plants treated with CaCl_2_ or 200 mM Ca(NO_3_)_2_ displayed signs of stunted growth and leaf yellowing resulting from decreased chlorophyll concentration (Figure 1A). Different chlorophyll *a* fluorescence-derived parameters showed some variation depending on the treatment, but in general, plants treated with CaCl_2_ or 200 mM Ca(NO_3_)_2_ had significantly lower values of fluorescence parameters (Figure 1B–D). Both Performance Index Inst (Figure 1C) as well as Performance Index Total (Figure 1D) were extremely sensitive indicators for calcium toxicity. In addition, while Performance Index Inst showed a tendency to increase in plants treated with NaCl, KCl or NaNO_3_ (Figure 1C), Performance Index Total showed a significant increase only in NaNO_3_-treated plants (Figure 1D).

**Figure 2:**
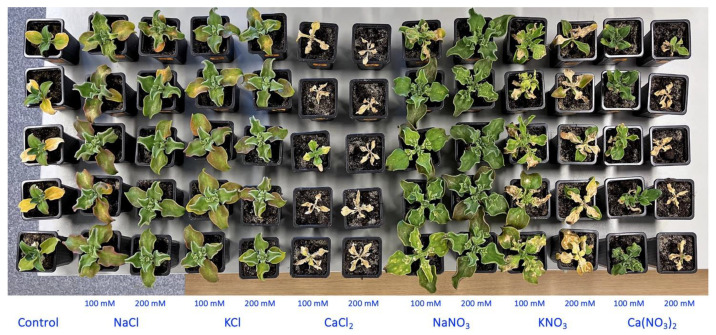
Overview of all *Mesembryanthemum crystallinum* plants used in the Experiment 1 at the end of the experiment.

**Figure 3:**
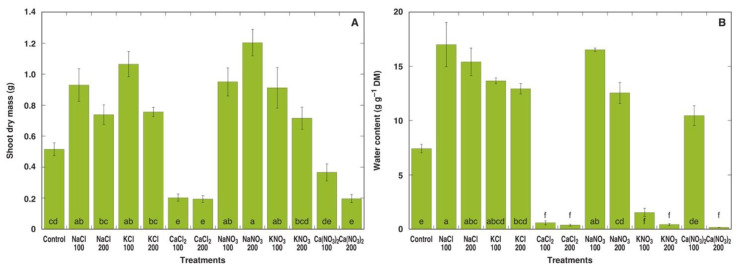
Effect of different salts on shoot dry mass (**A**) and water content in the fourth leaf pair (**B**) of *Mesembryanthemum crystallinum* plants. Numbers on x axis indicate respective salt concentration in soil (mM). Data are means from five replicates ± SE. Different letters indicate statistically significant differences according to the Tukey HSD test (*p* < 0.05).

Within the next two weeks, morphological differences between *M. crystallinum* plants under different treatments became more pronounced (Figure S2B,C). At the end of the experiment, all individual plants treated with 200 mM CaCl_2_ as well as four plants out of five for both the 100 mM CaCl_2_ and 200 mM Ca(NO_3_)_2_ treatments appeared dead, but plants in the 100 mM KNO_3_ and, to a larger extent, 200 mM KNO_3_ treatments showed signs of deterioration (Figure 2). Four weeks after the full treatment, this effect of the different salts was clearly visible in the dry biomass data (Figure 3A). While the shoot dry mass tended to increase for plants in all treatments with NaCl, KCl, NaNO_3_ and KNO_3_, only the values for 100 mM NaCl, 100 mM KCl, 100 and 200 mM NaNO_3_, and 100 mM KNO_3_ were statistically significantly different from the control. On the other hand, plants treated with 100 and 200 mM CaCl_2_ and 200 mM Ca(NO_3_)_2_ showed statistically significant decrease in dry shoot biomass. In addition, plants treated with NaCl, KCl and NaNO_3_ at both concentrations had significantly higher water content in shoots (Figure 3B). A significant decrease in the shoot water content to very low levels in plants treated with CaCl_2_, KNO_3_ or 200 mM Ca(NO_3_)_2_ reflected plant tissue death.

**Figure 4:**
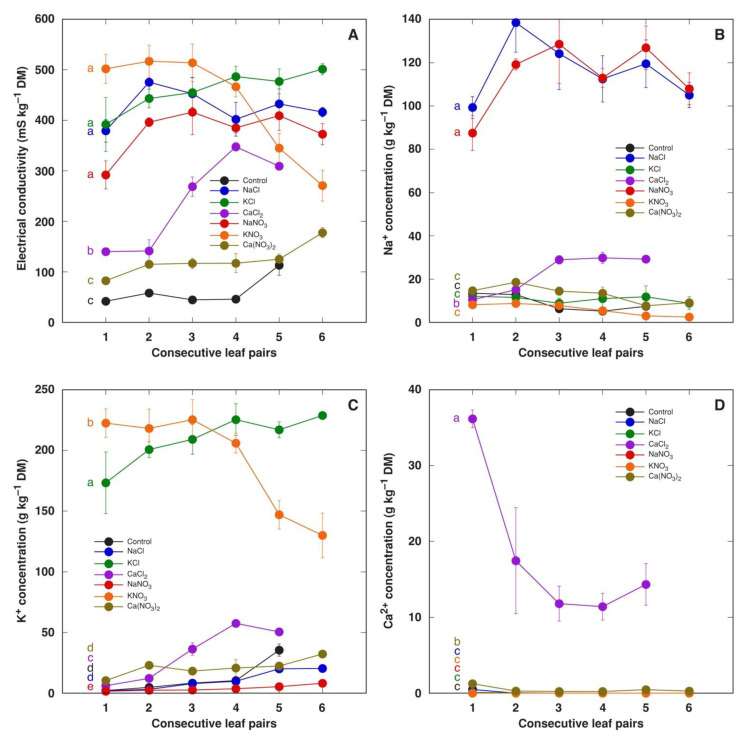
Effect of treatment with different salts on soluble ion content measured as electrical conductivity (**A**), Na^+^ concentration (**B**), K^+^ concentration (**C**) and Ca^2+^ concentration (**D**) in water extracts of different leaf pairs of *Mesembryanthemum crystallinum* plants. Data are means from measurement of six separate samples for each treatment/leaf pair combination ± SE. Both treatment concentrations were analyzed together. Different letters indicate statistically significant differences according to one-way ANOVA analysis (*p* < 0.05).

Water-soluble ion content in the leaves of *M. crystallinum* plants did not depend on the concentration of salts in the soil; therefore, the results were calculated as means from both concentrations for all salts. The total concentration of ions in leaf tissue water extracts in *M. crystallinum* leaf pairs of different ages was assessed by measuring EC (Figure 4A). In plants treated with NaCl, KCl, NaNO_3_ and KNO_3_, the EC level in tissue extracts was relatively similar. There was no gradient of EC in different leaf pairs except in plants treated with KNO_3_, which was associated with a lower accumulation potential for K^+^ in young leaves (Figure 4C). The level of EC was significantly lower in the leaves of plants treated with CaCl_2_ and, especially, Ca(NO_3_)_2_, indicating that nitrates were not present in a soluble form (Figure 4A). The accumulation capacity of Na^+^ was similar for NaCl- and NaNO_3_-treated plants (Figure 4B). Interestingly, both Na^+^ and K^+^ accumulation was stimulated in plants treated with CaCl_2_.

### 3.2. Experiment 2: Effect of Salinity Gradient of NaCl, KCl and CaCl_2_

**Figure 5:**
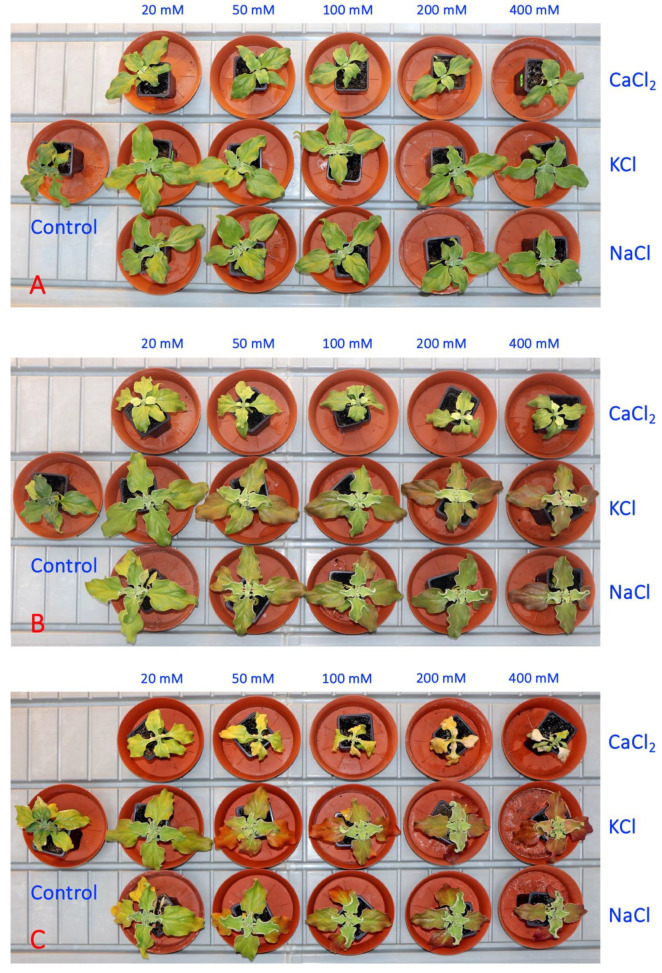
Representative *Mesembryanthemum crystallinum* plants used in the Experiment 2 one week (**A**), two weeks (**B**) and three weeks (**C**) after the last treatment.

In the second experiment, five concentrations each of NaCl, KCl and CaCl_2_ were used for the soil treatment of *M. crystallinum* plants. One week after the full treatment, there were visual signs of growth impairment in plants treated with a moderate or high concentration of CaCl_2_ in comparison to the growth of both NaCl- and KCl-treated plants (Figure 5A). Additional problems were clearly visible a week later, when CaCl_2_-treated plants started to loose chlorophyll which appeared as leaf yellowing (Figure 5B). Plant dieback at high CaCl_2_ concentrations (200 and 500 mM) started after another week (Figure 5C).

**Figure 6:**
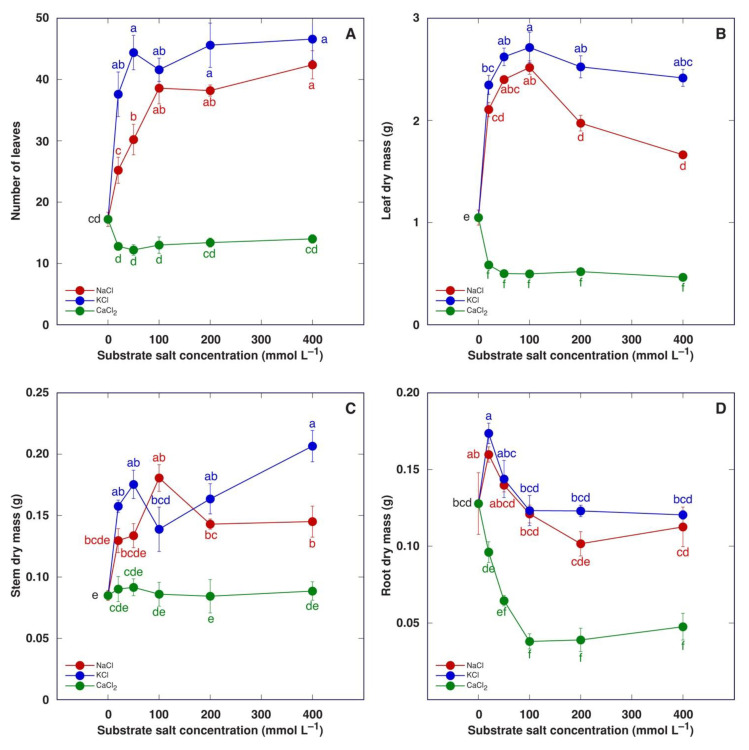
Effect of treatment with increasing concentration of different chloride salts on number of leaves (**A**), leaf dry mass (**B**), stem dry mass (**C**) and root dry mass (**D**) of *Mesembryanthemum crystallinum* plants. Data are means from five replicates ± SE. Different letters indicate statistically significant differences according to the Tukey HSD test (*p* < 0.05).

When the experiment was terminated four weeks after the full treatment, it was evident that treatment with NaCl or KCl had a positive effect on the number of leaves (Figure 6A) as well as dry mass of leaves (Figure 6B), stem (Figure 6C) and roots, but the concentration dependence of the effect was distinct. A stimulative effect on leaf development was clearly saturable with an increasing salt concentration, but root biomass increased by only 20 mM. Treatment with KCl tended to have higher stimulative effect in comparison to that with NaCl. Treatment with CaCl_2_ tended to decrease the number of leaves (Figure 6A) and significantly decrease the leaf dry mass (Figure 6B), but these effects were not concentration-dependent. The stem dry mass was not affected by CaCl_2_ treatment (Figure 6C), but the dry mass of the roots decreased in a concentration-dependent manner until it reached saturation at 100 mM (Figure 6D).

**Figure 7:**
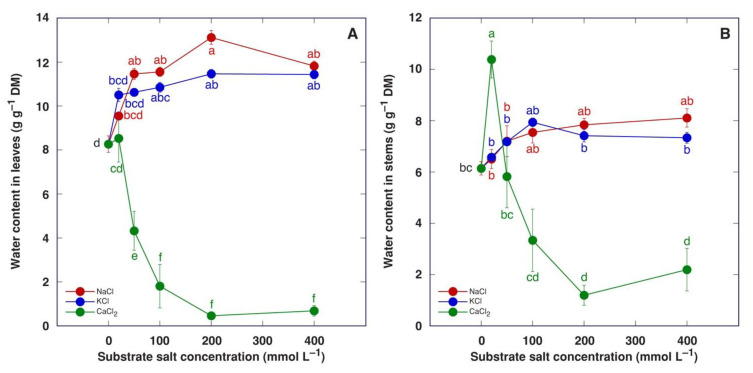
Effect of treatment with increasing concentration of different chloride salts on water content in leaves (**A**) and stems (**B**) of *Mesembryanthemum crystallinum* plants. Data are means from five replicates ± SE. Different letters indicate statistically significant differences according to the Tukey HSD test (*p* < 0.05).

Water content increased in both leaves and stems of *M. crystallinum* plants treated with NaCl or KCl (Figure 7). This effect was statistically significant for leaves starting from 50 mM NaCl and 100 mM KCl but not for stems. Treatment with CaCl_2_ caused a decrease in water content in leaves in a concentration-dependent manner (Figure 7A), but in stems, the 20 mM CaCl_2_ treatment resulted in a statistically significant increase in water content followed by a concentration-dependent decrease (Figure 7B).

**Figure 8:**
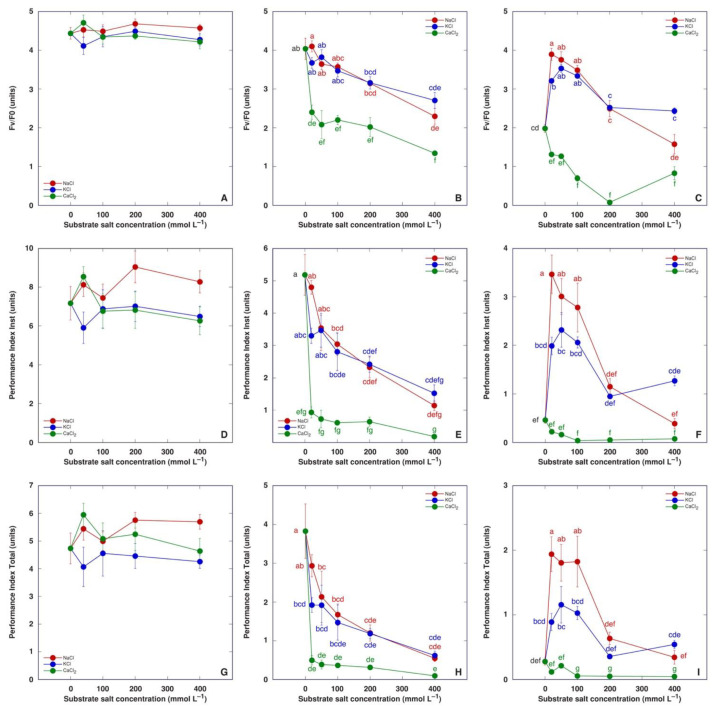
Effect of treatment with increasing concentration of different chloride salts on chlorophyll *a* fluorescence parameters, namely F_v_/F_0_ (**A**–**C**), Performance Index Inst (**D**–**F**) and Performance Index Total (**G**–**I**) of *Mesembryanthemum crystallinum* plants on the day of the last treatment (**A**,**D**,**G**), and one (**B**,**E**,**H**) and two (**C**,**F**,**I**) weeks after the last treatment. Data are means from five independent measurements ± SE. Different letters indicate statistically significant differences according to the Tukey HSD test (*p* < 0.05). There were no statistically significant differences for any parameters for plants before the last treatment (**A**,**D**,**G**).

Chlorophyll *a* fluorescence was measured three times during plant cultivation: on the day of the last treatment, and one and two weeks after the last treatment (Figure 8). No significant differences were evident in fluorescence parameters on the day of the last treatment (Figure 8A,D,G). One week later, all chlorophyll *a* fluorescence parameters in the leaves of salt-treated *M. crystallinum* plants decreased in a concentration-dependent manner—to the same degree as plants treated with NaCl or KCl, and more pronounced than CaCl_2_-treated plants (Figure 8B,E,H). One more week later, all chlorophyll *a* fluorescence parameters in the leaves of control plants had decreased, but those in plants treated with NaCl or KCl in the range of 20–100 mM showed significantly higher parameter values (Figure 8C,F,I). Plants treated with CaCl_2_ had the lowest values of chlorophyll *a* fluorescence parameters. Performance Index Total appeared to be the most sensitive indicator of the adverse effect of CaCl_2_.

The treatment with an increasing concentration of NaCl resulted in the concentration-dependent accumulation of Na^+^ in the leaves of *M. crystallinum* plants (Table 1). The dose–response characteristics showed the saturable nature of Na^+^ accumulation. Similar characteristics of accumulation were evident for K^+^ in KCl-treated plants. Moreover, the treatment with CaCl_2_ significantly stimulated both Na^+^ and K^+^ accumulation. The molar concentration ratio K^+^/Na^+^ significantly decreased in NaCl-treated plants, up to 50 mM salinity, with no changes with further increases in salinity (Table S1). However, this parameter did not significantly change in CaCl_2_-treated *M. crystallinum* plants. The accumulation of Cl^−^ was slightly more pronounced in KCl-treated plants in comparison to the ones treated with NaCl, but the accumulation of Cl^−^ in CaCl_2_-treated plants was significantly lower at 200 and 400 mM. The accumulation of calcium in CaCl_2_-treated plants also had characteristics of saturation, but treatment with NaCl or KCl had no effect on the calcium concentration in leaves.

The concentration of macronutrient phosphorus significantly decreased in plants treated with KCl or NaCl and increased in plants treated with CaCl_2_ (Table 1). A similar effect was evident for the macronutrient magnesium, but the stimulatory effect of CaCl_2_ was statistically significant only in 20–50 mM treatments. For the micronutrients iron and manganese, treatment with NaCl or KCl resulted in a decrease in their concentration in leaves, but the effect was not statistically significant for all concentrations used. However, CaCl_2_ treatment had no significant effect on the concentration of iron or manganese. The concentration of micronutrient zinc significantly decreased in plants treated with NaCl or KCl, and significantly increased in plants treated with CaCl_2_. For micronutrient copper, treatment with NaCl or KCl tended to decrease its concentration, but the effect was not always statistically significant. Moreover, the concentration of copper significantly increased in plant leaves treated with several concentrations of CaCl_2_.

**Figure 9:**
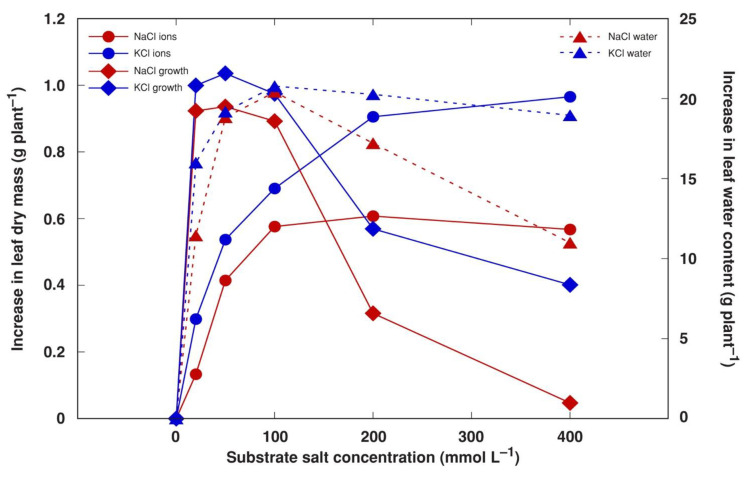
Calculated effect of increase in the accumulation of soluble ions (Na^+^, K^+^ and Cl^−^), growth-related increase in dry biomass and increase in water content in stimulation of biomass accumulation by NaCl and KCl treatment with increasing salt concentration in leaves of *Mesembryanthemum crystallinum* plants in comparison to control levels.

To characterize the investment of different biomass components in the overall positive growth response in NaCl- and KCl-treated plants, the amount of accumulated Na^+^, K^+^ and Cl^−^ in leaves was calculated on an individual plant basis and compared to the increase in dry biomass minus the mass of these ions, possibly reflecting the increase in dry organic matter. It appeared that the optimum range for an increase in organic biomass production was 20–100 mM of salt in the soil for both NaCl and KCl (Figure 9). However, the maximum capacity for ion accumulation was reached at 100 mM NaCl and 200 mM KCl. In addition, the contribution of water accumulation to fresh biomass peaked at 50 mM salinity and tended to decline as salinity increased, especially, for NaCl-treated plants.

## 4. Discussion

The critical feature of obligatory halophytic plant species is their dependence on an increased rootzone salt concentration for optimum growth [48]. The results of the present study clearly indicated *M. crystallinum* as an obligatory halophyte species, as plant growth was significantly stimulated by both NaCl and KCl treatments (Figure 3A and Figure 6). Similarly, the optimum growth of *M. crystallinum* plants occurred at 100–200 mM NaCl in irrigation water, and no decrease in biomass accumulation was evident even at 800 mM NaCl [39]. However, not all studies confirm the obligatory halophytic nature of *M. crystallinum*. When 40-day-old vermiculite-cultivated plants were treated with 100 mM NaCl for two weeks, a significant reduction in growth was evident [21]. However, no significant effect from a similar treatment was seen for the treatment of 55-day-old plants. In another study using vermiculite-cultivated *M. crystallinum* plants, the leaf fresh mass and leaf area increased at 100 mM NaCl within 15 days, but shoot dry mass did not change in saline conditions, indicating that only water accumulation was promoted [49].

Components of salinity-induced biomass increases in obligatory halophytes have been analyzed previously [50]. It has been argued that increases in biomass in the majority of halophytes under increased salinity is mostly due to the accumulation of mineral ions and water [50,51], although, at least for some species, an increase in organic dry mass at increased salinity levels has been documented. In the present study, the optimum organic biomass accumulation for *M. crystallinum* plants occurred at 20–100 mM NaCl or KCl (Figure 9), but the total dry mass was still higher than control values at 400 mM salinity (Figure 6B) due to a large amount of accumulated ions. However, in another study, the fresh mass of *M. crystallinum* plants treated with 400 mM NaCl was similar to that of control plants, but the dry mass significantly decreased at this salinity level [13]. Similar to the results of the present study, the stem-succulent hydrohalophyte species *Arthrocnemum macrostachyum* showed an optimum dry biomass increase at 200–400 mM NaCl, and this increase was due to the accumulation of both organic biomass as well as mineral elements [40].

Ionic requirements for the increased growth of halophytic species under salinity had not been much studied. Halophytic legume species *Prosopis strombulifera* showed optimum growth at 150–300 mM NaCl, but treatment with Na_2_SO_4_ did not show any stimulative effect on plants [31]. The growth of *Atriplex gmelini* was stimulated by addition of 50 mM NaCl in the culture solution, and the same effect was achieved by the addition of 50 mM KCl, 25 mM Na_2_SO_4_, or 25 mM K_2_SO_4_ [30]. However, at 100 mM, both NaCl and KCl resulted in a stimulatory effect, while treatment with MgCl_2_ had no effect on plant growth. The growth of the stem-succulent hydrohalophyte *Salicornia europaea* was optimal at 200 mM NaCl or NaNO_3_, while at 200 mM KNO_3_, it was less efficient in growth stimulation, but 200 mM KCl inhibited plant growth [52]. In the present study, for *M. crystallinum* plants, the effect of sodium and potassium in the form of chloride salts for growth stimulation was rather similar. However, the type of anion significantly modified the effect of these cations, as NaNO_3_ was more efficient in the stimulation of growth and development in comparison to chlorides, but KNO_3_, while showing some initial stimulatory eventually effect caused plant deterioration. Moreover, when calcium was provided in the form of nitrate, the treatment had fewer negative consequences in comparison to those caused by calcium chloride, especially at low concentrations. These results are, in principle, consistent with previous observations that the effects of sodium and potassium salts depend significantly on the type of anion, which is related to the different effects of these anions on plant metabolism [35,36]. In this case, the stimulating effect of nitrate on plant growth and development could be related to the nitrophilic nature of *M. crystallinum* plants. Similarly, treatment with sodium in the form of nitrate resulted in a positive effect on plants, for which growth was reduced by sodium chloride [36].

The mechanisms of plant growth promotion by sodium and potassium in obligatory halophytic species are still under scientific debate. Since the early studies, the accumulation of both Na^+^ and Cl^−^ in shoot tissues are suggested to provide necessary osmotic potential in cells, allowing for water uptake and further increasing cell turgor and cell wall elasticity, resulting in cell elongation [50,53]. However, a direct comparison between the effects of NaCl and those of non-ionic osmotica has led to the conclusion that cell growth stimulation in halophytes is related to the ionic rather than osmotic effect of NaCl [13,54]. Furthermore, direct hormone-mediated growth stimulation effects in halophytes at increased salinity levels cannot be excluded, as these are related to the action of endogenous ethylene [55].

The most surprising finding in this study was the strongly negative effect of CaCl_2_, which appeared at the level of both metabolic regulation as the reduced photochemical activity of photosynthesis in the early response phase and as growth retardation a little later, followed by the complete dieback of plants. Therefore, it seems that *M. crystallinum* responds to increased substrate calcium as would a typical calcifuge species similar to the ones occurring natively in acidic soils [56]. The presence of calcium is generally known to have a positive effect on plant salt tolerance, especially for glycophytes [37]. Moreover, salt tolerance in halophyte species has been shown to be positively affected by a moderate calcium concentration (20 mM), as in *Cakile maritima* cultivated in conditions of sand hydroponics [57]. In particular, the restoration of plant growth under moderate and high NaCl (200 and 400 mM) was associated with the improved status of enzymatic antioxidants and lowered levels of indicators of oxidative stress. However, in the monocotyledonous halophyte species *Triglochin maritima*, treatment with a moderate level of NaCl (4 g kg^−1^ Na^+^) resulted in increased shoot growth, but the addition of CaCO_3_ (14 g Ca^2+^ kg^−1^) in soil completely abolished this effect, together with a drastic decrease in Na^+^ accumulation in tissues [58].

In general, calcium is considered to be non-toxic for plants, but it is suggested that excessive levels of calcium in soil can unfavorably affect the uptake of other nutrients [56]. The uptake of potassium, magnesium, and iron was shown to be negatively affected by excess soil calcium concentration, resulting in the deficiency of the respective mineral elements [59]. However, calcium toxicity in the present study was not related to mineral deficiency, as there was no negative effect on mineral nutrition, and the concentration of several macronutrients (phosphorus and magnesium) and micronutrients (zinc and copper) was significantly increased in CaCl_2_-treated plants (Table 1).

The obtained results of growth inhibition in *M. crystallinum* plants by calcium salts clearly contradict those presented by Madhavi et al. 2022 [18], showing that treatment with CaCl_2_ resulted in the growth stimulation of *M. crystallinum* plants cultivated both at low and moderate salinity. It is possible that such significant differences could be attributed to differences in experimental conditions and/or plant genotype characteristics. Unfortunately, the source of seeds was not indicated in this study, but plants in the experimental stage were cultivated in 14.4 L containers, with three plants per container. In contrast, individual containers with a limited substrate volume (0.2 L) were used in the present study. Another difference was related to the light quantity and quality: red-, blue- and green-light emitting diodes with a 120 μmol m^−2^ s^−1^ photon flux density of photosynthetically active radiation (16 h photoperiod) were used in the study of Madhavi et al. 2022 [18], while in the present study, natural light with additional illumination at a 380 μmol m^−2^ s^−1^ photon flux density of photosynthetically active radiation (16 h photoperiod) was used. Additional differences were related to the salt treatment: In the study by Madhavi et al. 2022 [18], 14.4 L containers were treated daily with 25 mL of fertilizer solution plus 25 mL of a salt solution of varying concentrations, but the final amount of applied salts or substrate EC level at the end of the experiment were not indicated. Theoretically, during the three months of the salt treatment, each container would have received 900 mmol of salt in the case of a 400 mM salt solution treatment, resulting in a final salinity of 62.5 mmol L^−1^, with a daily increase in salinity of only 0.694 mmol per L of substrate. In contrast, in the present study, the salt treatment was performed in 50 mmol L^−1^ increments twice a week, which is distinctly different from the one described above. As it is well known that the salt treatment procedure used in that particular experiment significantly affects the response of plants to salinity [60], it is highly likely that differences in the of salt treatment procedure between the two experiments were among the factors leading to the contradictory results.

*M. crystallinum* has been described as a salt-accumulating type of halophyte with a low root concentration of Na^+^ and with a further increase in stems, leaves and, especially, epidermal bladder cells [8]. The high sodium accumulation ability in shoots of *M. crystallinum* plants has been shown to be due to the high activity of two tonoplast transport systems, Na^+^/H^+^ antiport and H^+^ translocating V-ATP-ase, resulting in the efficient vacuolar sequestration of Na^+^ [61]. A more detailed study using suspension-cultured cells of *M. crystallinum* has revealed that the expression of a group of ion homeostasis-related genes positively correlated with growth activation in saline conditions [13]. These included both plasma membrane transporters and channels (for nitrate, sodium, potassium and cations/chloride) as well as tonoplast antiporters (H^+^/Cl^−^ and Na^+^/H^+^) and V-ATP-ase.

However, the particular ion accumulation capacity seems to be highly variable between different experiments, clearly pointing to genetical variability or differences in experimental conditions. Surprisingly, the control plants of *M. crystallinum* accumulated up to 100 g kg^−1^ of Na^+^ in their leaves at the adult stage when fertilized with Hoagland solution only; even the treatment with 400 mM NaCl did not result in increased Na^+^ accumulation, but it was significantly stimulated by treatment with 200 or 400 mM CaCl_2_ alone [18]. In another study, control plants accumulated up to 55 g kg^−1^ Na^+^, and this value increased up to 140 g kg^−1^ under salinity, but the effect was not concentration-dependent [22]. In contrast, in the present study, the accumulation of both Na^+^ (in NaCl-treated plants) and K^+^ (in KCl-treated plants) initially was concentration-dependent, but reached near-saturation level at 200 mM salinity (Table 1).

Mineral nutrition problems have been mentioned as one of the reasons for reduced plant growth under salinity conditions. Among mineral elements, due to chemical similarity between sodium and potassium, the retention of adequate potassium concentration in leaf mesophyll has been suggested as one of mechanisms of salinity tolerance in halophytes, including *M. crystallinum* [62]. However, a general relationship between changes in potassium concentration in saline environments and salt tolerance in plants native to saline habitats has not been demonstrated. While in some halophytes, NaCl treatment results in a significant decrease in shoot K^+^ concentration [32], in *M. crystallinum* plants, the K^+^ concentration tended to decrease at low NaCl salinity levels, but this effect was not statistically significant (Table 1). When different accessions of the relatively salt-tolerant legume species *Trifolium fragiferum* were compared, the K^+^ concentration in leaf blades increased only at high salinity levels in four accessions out of six, while one accession showed no changes, and for one accession, K^+^ increased already at low salinity levels [38].

Results from studies with obligatory halophyte species show rather variable responses of mineral nutrition to increasing salinity. In the stem-succulent euhalophyte *Arthrocnemum macrostachyum*, increased NaCl up to 600 mM results in significantly reduced shoot concentration of calcium, magnesium and potassium [40]. In leaves of *Prosopis strombulifera*, NaCl treatment did not affect the potassium concentration but significantly reduced that of calcium [31]. For *M. crystallinum*, some information can be found on the effects of increased salinity on mineral nutrition. Thus, it was shown that increasing salinity progressively decreased the accumulation of the macronutrients phosphorus, potassium, calcium and magnesium in plant leaves, while the accumulation of micronutrients were either unaffected (manganese and iron) or only temporarily decreased (zinc and copper) in a situation when salinity resulted in increased plant growth [22]. In a study with *M. crystallinum* plants on the background of low-salinity-induced growth and with no negative effects of high salinity, increasing NaCl resulted in a significant reduction in shoot nitrogen, phosphorus, sulfur, magnesium and potassium, but micronutrient levels were not significantly affected [39]. In another study where no stimulative effect of salinity on growth was found, NaCl treatment resulted in a significant decrease in leaf phosphorus and potassium, but changes in the concentration of calcium, magnesium, manganese, zinc and iron were not unambiguous [49]. In the present study, there was a tendency that mineral nutrient concentrations in leaves of *M. crystallinum* decreased both in NaCl- and KCl-treated plants (Table 1). Therefore, it is highly likely that the observed effects on leaf mineral content due to different salts are directly related to growth changes caused by these salts. Assuming that root uptake for a particular element remains constant, factors that cause increased biomass accumulation (such as NaCl and KCl in this particular case) should result in a reduced element concentration, but factors leading to growth reduction (such as CaCl_2_ in this particular case) should lead to an increased element concentration. A similar “mineral dilution effect” has been described previously and has been proven experimentally in various research systems [63,64,65].

In some earlier studies, differences between the photochemical activity of photosystem II between control and salt-treated *M. crystallinum* plants have been associated with a transfer from a C_3_- to a CAM-type of photosynthesis [66]. In particular, the non-photochemical component of fluorescence quenching was higher with the transition to CAM. On the other hand, moderate salinity can improve the photochemistry of photosynthesis in *M. crystallinum* plants at the level of linear electron transport [67]. In another study, salt-treated *M. crystallinum* plants (150 mM NaCl) had significantly higher F_v_/F_m_ and saturated photosynthetic electron transport rates in comparison to those of control plants [15]. However, it was shown that at 400 mM NaCl, acute photoinhibition occurred, which was further amplified in high light (1000 μmol m^−2^ s^−1^) conditions [27]. Moreover, photochemical quenching decreased and non-photochemical quenching increased at suboptimal salinity (500 mM NaCl) for hydroponically cultivated *M. crystallinum* plants [19].

It was not assessed whether CAM was induced in *M. crystallinum* plants by any of the treatments in the present study, but it has been suggested that CAM becomes gradually inducible only within the adult stage of *M. crystallinum* plants, when side shoots with secondary leaves are produced in parallel to the senescence of primary leaves [9]. Therefore, it is highly likely that plants were at the C_3_ stage throughout the experiment, and that the measured changes in the photochemistry of photosynthesis reflect direct salinity effects, most likely related to general metabolic stimulation.

Recorded changes in chlorophyll *a* fluorescence parameters confirmed that the response of *M. crystallinum* plants to treatment by different salts was extremely fast. On the day of the last treatment no statistically significant differences in any of the parameters were found between the different treatments (Figure 8A,D,G). Only one week later, salinity treatments resulted in a concentration-dependent decrease for all fluorescence-derived parameters (Figure 8B,E,H), but after one more week, values of fluorescence parameters decreased in control plants and plants treated with 400 mM NaCl or KCl, but not in plants treated with NaCl or KCl with a lower concentration of salts (Figure 8C,F,I). Both Performance Index Inst and Performance Index Total were very sensitive indicators of salinity-induced metabolic changes in plants. Only Performance Index Total positively responded to nitrate salts (Figure 1D), showing that the particular parameter is less useful in directly evaluating salinity effects. As Performance Index Total is sensitive also to alterations in photochemical reactions at the donor side of photosystem II, such as the activity of a water-splitting complex [42,68], it reflects changes in physiological performance due to changes in mineral nutrient availability [69,70].

The significant differences in the experimental results obtained in different studies regarding the response of *M. crystallinum* to salinity could be at least partly due to the pronounced adaptive plasticity of these plants. Thus, the species belongs to the plants that can adapt their individual size to nutrient availability in the soil and/or volume for root growth [8]. In addition, the reaction of the plant to changes in environmental conditions, including salinity, depends on the specific development stage the plant is in. On the other hand, development can happen slower or faster depending on the set of conditions. For this reason, pre-defined development phases should be followed during research. The development of small secondary leaves on side shoots in addition to up to seven pairs of large primary leaves on the main rosette are indicative of the transition to the adult phase of the individual, i.e., when it gradually becomes competent for the induction of CAM [9].

## 5. Conclusions

Sodium and potassium in the form of chloride salts had a very similar positive effect on the growth and physiological status of *M. crystallinum* plants. Moreover, sodium nitrate was more efficient in the stimulation of growth and development in comparison to chloride, but potassium nitrate, while showing some initial stimulatory effect, eventually caused plant deterioration. Calcium salts showed pronounced toxicity symptoms, which manifested itself as growth retardation, a decrease in the photochemical activity of photosynthesis, the loss of chlorophyll and plant dieback, but CaCl_2_ was more harmful in comparison to Ca(NO_3_)_2_. Changes in leaf mineral nutrient concentration showed a typical “dilution effect” (decrease in concentration) in a situation when plant growth was stimulated by NaCl or KCl, and increased mineral element concentration when plant growth was inhibited by CaCl_2_.

## Figures and Tables

**Table 1:** Effect of salinity on mineral element concentration in leaves of *Meembryanthemum crsytallinum* plants.

Treatment	Na (g kg^−1^)	K (g kg^−1^)	Cl (g kg^−1^)	Ca (g kg^−1^)	P (g kg^−1^)	Mg (g kg^−1^)	Fe (mg kg^−1^)	Mn (mg kg^−1^)	Zn (mg kg^−1^)	Cu (mg kg^−1^)
Control	3.1 ± 0.1 h	16.7 ± 0.1 g	1.8 ± 0.1 i	8.1 ± 0.4 d	4.3 ± 0.6 c	7.0 ± 0.5 bc	111 ± 15 abc	253 ± 31 ab	94 ± 9 b	8.1 ± 0.3 cde
NaCl 20 mM	12.1 ± 0.3 fgh	8.3 ± 0.5 g	53.8 ± 1.9 h	5.0 ± 0.2 d	2.5 ± 0.0 de	3.3 ± 0.2 de	67 ± 4 de	166 ± 12 cd	45 ± 1 cd	4.2 ± 0.3 g
NaCl 50 mM	86.6 ± 3.2 d	7.4 ± 1.0 g	88.3 ± 2.2 ef	5.3 ± 1.1 d	2.2 ± 0.1 e	2.9 ± 0.4 de	87 ± 12 bcd	123 ± 6 cd	33 ± 1 d	3.9 ± 0.2 g
NaCl 100 mM	125.1 ± 4.1 c	8.5 ± 0.2 g	105.8 ± 6.8 de	6.3 ± 0.2 d	2.5 ± 0.1 de	3.3 ± 0.5 de	73 ± 3 cde	163 ± 13 cd	36 ± 4 cd	5.3 ± 1.1 fg
NaCl 200 mM	158.2 ± 3.2 b	10.7 ± 0.4 g	152.5 ± 3.8 c	5.2 ± 0.3 d	2.8 ± 0.1 de	2.4 ± 0.1 e	73 ± 10 cde	165 ± 26 cd	40 ± 1 cd	6.2 ± 0.4 efg
NaCl 400 mM	179.2 ± 5.6 a	11.3 ± 1.9 g	165.0 ± 4.3 bc	4.8 ± 0.3 d	3.4 ± 0.1 cd	2.3 ± 0.1 e	62 ± 7 de	148 ± 23 cd	53 ± 3 c	6.1 ± 0.7 efg
KCl 20 mM	2.2 ± 0.1 h	80.5 ± 2.3 de	55.0 ± 2.2 h	5.7 ± 0.4 d	1.7 ± 0.1 e	3.2 ± 0.2 de	62 ± 4 de	106 ± 6 cd	41 ± 5 cd	5.1 ± 0.3 fg
KCl 50 mM	2.1 ± 0.2 h	126.1 ± 2.5 c	86.3 ± 4.0 f	9.1 ± 2.0 d	1.9 ± 0.0 e	4.2 ± 0.7 d	76 ± 4 cde	178 ± 16 bc	53 ± 1 c	7.4 ± 0.5 def
KCl 100 mM	3.5 ± 0.3 h	146.5 ± 5.2 b	115.0 ± 2.5 d	6.9 ± 0.2 d	2.0 ± 0.1 e	3.0 ± 0.1 de	71 ± 11 de	137 ± 12 cd	51 ± 3 c	6.4 ± 0.4 efg
KCl 200 mM	6.0 ± 0.2 gh	195.6 ± 5.4 a	170.8 ± 3.3 b	9.6 ± 0.6 d	2.0 ± 0.1 e	3.4 ± 0.1 de	51 ± 3 de	140 ± 13 cd	42 ± 1 cd	6.1 ± 0.2 efg
KCl 400 mM	5.4 ± 1.1 h	205.9 ± 5.0 a	201.7 ± 7.3 a	8.8 ± 0.9 d	2.1 ± 0.0 e	3.1 ± 0.1 de	51 ± 6 e	92 ± 4 d	43 ± 3 cd	4.3 ± 0.3 g
CaCl_2_ 20 mM	9.7 ± 0.3 gh	39.8 ± 0.2 f	63.8 ± 1.4 gh	34.4 ± 0.6 c	6.3 ± 0.2 b	11.4 ± 0.0 a	147 ± 0 b	305 ± 15 a	128 ± 4 a	10.3 ± 1.1 abc
CaCl_2_ 50 mM	12.7 ± 0.1 fgh	67.5 ± 1.9 e	81.9 ± 0.4 fg	54.1 ± 0.1 b	7.2 ± 0.3 ab	10.8 ± 0.1 a	139 ± 2 ab	308 ± 10 a	138 ± 2 a	8.9 ± 0.1 abcd
CaCl_2_ 100 mM	16.4 ± 0.1 efg	79.9 ± 2.8 de	91.9 ± 3.3 ef	71.4 ± 3.1 a	7.4 ± 0.5 ab	8.5 ± 0.1 b	119 ± 4 ab	289 ± 6 a	134 ± 3 a	10.2 ± 0.5 bcde
CaCl_2_ 200 mM	21.2 ± 0.1 ef	90.0 ± 1.7 d	100.0 ± 0.7 def	77.8 ± 6.2 a	7.9 ± 0.0 a	7.2 ± 0.3 bc	132 ± 4 a	276 ± 1 a	133 ± 3 a	11.1 ± 0.6 ab
CaCl_2_ 400 mM	25.0 ± 0.2 e	88.0 ± 6.4 d	111.3 ± 2.2 d	76.1 ± 1.5 a	7.1 ± 0.0 ab	6.6 ± 0.2 c	137 ± 7 ab	293 ± 13 a	144 ± 1 a	12.9 ± 0.1 a

Data are means from three replicates ± SE. Different letters indicate statistically significant differences according to the Tukey HSD test (*p* < 0.05). For sake of comparison, values in red indicate statistically significant increases compared to control values, and values in blue indicate statistically significant decreases.

## Data Availability

All data reported here are available from the authors upon request.

## Supplementary Materials

The following supporting information can be downloaded at: https://www.mdpi.com/article/10.3390/plants13020190/s1, Figure S1: Representation of sequential leaf pairs of typical control plants of *Messembryanthemum crystallinum* used in the Experiment 1; Figure S2: Representative *Messembryanthemum crystallinum* plants used in the Experiment 1; Table S1: Effect of salinity on K^+^/Na^+^ molar concentration ratio in leaves of *Messembryanthemum crystallinum* plants.
