# Supplementary material for: Effect of Na, K and Ca Salts on Growth, Physiological Performance, Ion Accumulation and Mineral Nutrition of Mesembryanthemum crystallinum
Source: Plants (Basel). 2024 Jan 10;13(2):190. doi: 10.3390/plants13020190 (PMC10818879; doi:10.3390/plants13020190)
Supplement: Supplementary file 1 [file plants-13-00190-s001.zip › plants-2817308-supplementary.pdf]

Article

Effect of Na, K and Ca Salts on Growth, Physiological Performance, Ion Accumulation and Mineral Nutrition of *Mesembryanthemum crystallinum*

Astra Jēkabsone [1], Andis Karlsons [2], Anita Osvalde [2] and Gederts Ievinsh [1,*]

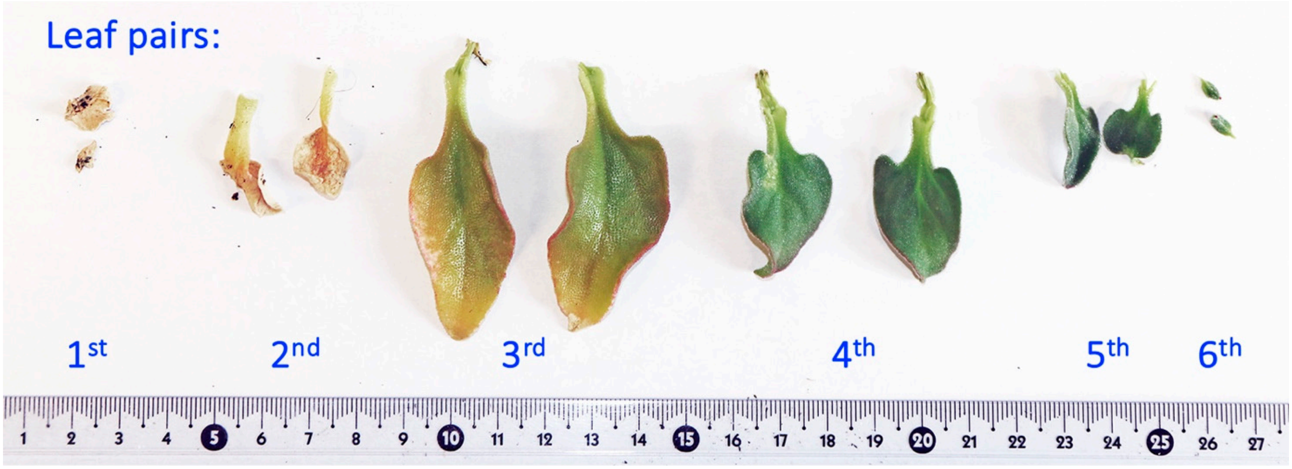

Figure S1. Representation of sequential leaf pairs of typical control plants of *Messembryanthemum crystallinum* used in the Experiment 1.

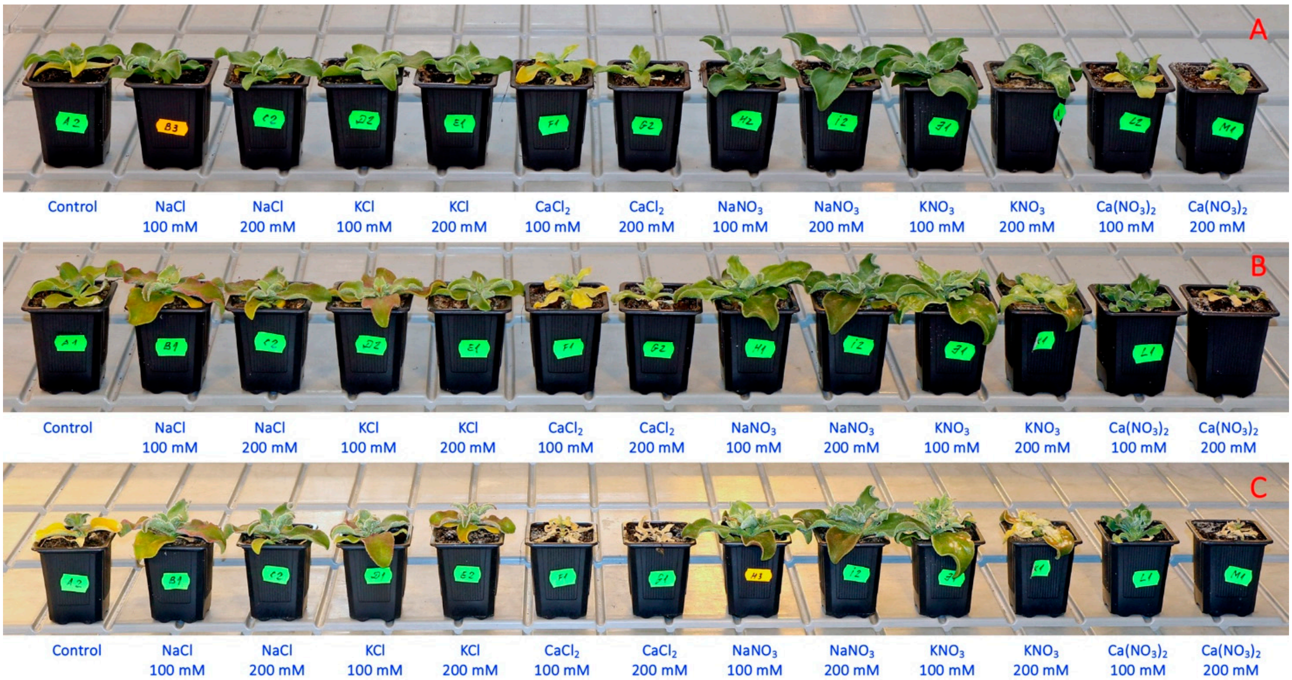

Figure S2. Representative *Messembryanthemum crystallinum* plants used in the Experiment 1: one week after the full treatment (A), two weeks after the full treatment (B), and three weeks after the full treatment (C).

Table S1. Effect of salinity on K^+/Na^+ molar concentration ratio in leaves of *Messembryanthemum crystallinum* plants.

**Treatment**	**NaCl**	**KCl**	**$CaCl_2$**
0 mM	3.160 ± 0.077 c	3.2 ± 0.1 c	3.16 ± 0.08 c
20 mM	0.405 ± 0.025 d	21.4 ± 1.0 b	2.42 ± 0.06 c
50 mM	0.050 ± 0.005 e	36.3 ± 3.6 a	3.15 ± 0.08 c
100 mM	0.040 ± 0.001 e	25.1 ± 2.2 b	2.87 ± 0.11 c
200 mM	0.040 ± 0.002 e	19.3 ± 1.1 b	2.50 ± 0.04 c
400 mM	0.037 ± 0.005 e	24.2 ± 3.9 b	2.08 ± 0.17 c

Data are means from three replicates ± SE. Different letters indicate statistically significant differences according to the Tukey HSD test ($p < 0.05$).
